# Forensic odontology: Assessing bite wounds to determine the role of teeth in piscivorous fishes

**DOI:** 10.1093/iob/obac011

**Published:** 2022-03-12

**Authors:** Pooventhran Muruga, David R Bellwood, Michalis Mihalitsis

**Affiliations:** Research Hub for Coral Reef Ecosystem Functions, James Cook University, Townsville, QLD 4811, Australia; College of Science and Engineering, James Cook University, Townsville, QLD 4811, Australia; Australian Research Council Centre of Excellence for Coral Reef Studies, James Cook University, Townsville, QLD 4811, Australia; Research Hub for Coral Reef Ecosystem Functions, James Cook University, Townsville, QLD 4811, Australia; College of Science and Engineering, James Cook University, Townsville, QLD 4811, Australia; Australian Research Council Centre of Excellence for Coral Reef Studies, James Cook University, Townsville, QLD 4811, Australia; Research Hub for Coral Reef Ecosystem Functions, James Cook University, Townsville, QLD 4811, Australia; College of Science and Engineering, James Cook University, Townsville, QLD 4811, Australia; Australian Research Council Centre of Excellence for Coral Reef Studies, James Cook University, Townsville, QLD 4811, Australia

## Abstract

Teeth facilitate the acquisition and processing of food in most vertebrates. However, relatively little is known about the functions of the diverse tooth morphologies observed in fishes. Piscivorous fishes (fish-eating fish) are crucial in shaping community structure and rely on their oral teeth to capture and/or process prey. However, how teeth are utilized in capturing and/or processing prey remains unclear. Most studies have determined the function of teeth by assessing morphological traits. The behavior during feeding, however, is seldom quantified. Here, we describe the function of teeth within piscivorous fishes by considering how morphological and behavioral traits interact during prey capture and processing. This was achieved through aquarium-based performance experiments, where prey fish were fed to 12 species of piscivorous fishes. Building on techniques in forensic odontology, we incorporate a novel approach to quantify and categorize bite damage on prey fish that were extracted from the piscivore’s stomachs immediately after being ingested. We then assess the significance of morphological and behavioral traits in determining the extent and severity of damage inflicted on prey fish. Results show that engulfing piscivores capture their prey whole and head-first. Grabbing piscivores capture prey tail-first using their teeth, process them using multiple headshakes and bites, before spitting them out, and then re-capturing prey head-first for ingestion. Prey from engulfers sustained minimal damage, whereas prey from grabbers sustained significant damage to the epaxial musculature. Within grabbers, headshakes were significantly associated with more severe damage categories. Headshaking behavior damages the locomotive muscles of prey, presumably to prevent escape. Compared to non-pharyngognaths, pharyngognath piscivores inflict significantly greater damage to prey. Overall, when present, oral jaw teeth appear to be crucial for both prey capture and processing (immobilization) in piscivorous fishes.

## Introduction

The development of a biting jaw within gnathostomes has long been hailed as a significant innovation in the evolution of vertebrates (Mallatt 1996; Kuratani 2012). While various uses have been documented for jaws, they are primarily associated with feeding, allowing for a wide range of food resources to be exploited (Clark and Summers 2007). To utilize diverse trophic niches, specialized teeth have developed within the jaws, facilitating prey capture, retention, and/or food processing (Frazzetta 1988; Anderson and LaBarbera 2008; Hocking et al. 2016). Given that the success of extant lineages is largely predicated on the ability of individuals to successfully acquire food (Ungar 2010; Wainwright et al. 2015; Konow et al. 2017), it is important to examine the feeding abilities (i.e., performance) of vertebrates.

The performance of an individual during specific tasks (e.g., feeding) is governed primarily by its phenotype (Wainwright and Reilly 1994). Studies of functional morphology have helped elucidate relationships between morphological traits and an individual's feeding performance (Norton and Brainerd 1993; Day et al. 2015; Galloway et al. 2016). These empirically derived relationships are subsequently used to infer broader morphology-based trophic links across various other taxa (Wainwright and Richard 1995). However, compared to terrestrial-based feeding, aquatic-based feeding poses fundamentally different challenges (Ferry et al. 2015; Wainwright et al. 2015). With fishes accounting for approximately half of all extant vertebrate species (Eschmeyer et al. 2010), understanding the feeding mechanisms surrounding this highly speciose group continues to garner interest today. Quantitative biomechanical models have proven particularly useful in describing and predicting how fishes use their oral jaws during feeding (Muller et al. 1982; Westneat 2003). More recently, such a comparative framework has been utilized to identify the function of teeth within fishes through quantitative measurements of tooth morphology (Anderson and LaBarbera 2008; Cohen, Weller, and Summers 2020; Cohen et al. 2020). These studies have been invaluable in providing an understanding of how morphological traits can provide clues relating to the life history of animals in the wild. However, there is still a need to test these hypotheses in performance-based experiments to causally link morphology to behavior (Wainwright and Reilly 1994).

Piscivorous fishes (i.e., fishes feeding predominantly on other fishes) are pivotal in structuring reef fish assemblages and maintaining trophic links within these ecosystems (Graham et al. 2003; Boaden and Kingsford 2015; Hixon 2015). With up to 53% of fishes on coral reefs capable of piscivory (Randall 1967; Hixon 1991), these fishes are able to shape coral reef ecosystems through predation. Yet, despite feeding on prey that are generally soft-bodied, piscivorous fishes display a remarkable diversity of feeding morphologies and strategies (Wainwright and Bellwood 2002; Mihalitsis and Bellwood 2019a), with the oral jaws serving as the primary mode of prey capture in piscivorous fishes (Liem 1980). Given this reliance on the oral jaws, an understanding of the functional basis of food acquisition in these fishes is critical in revealing the ecosystem role of piscivorous fishes in aquatic ecosystems.

When capturing elusive prey, some piscivorous fishes rely on their teeth to grab and retain these prey items (Hoyle and Keast 1988; Nanami and Shimose 2013; Galloway et al. 2016). Thus, a vast majority of studies have focused on the morphology of the teeth and the oral jaws. These studies have identified several key traits believed to affect prey capture, with oral gape, bite force, bite velocity, tooth shape, size, and arrangement regarded as major factors influencing feeding performance (Porter and Motta 2004; Habegger et al. 2011; Ferguson et al. 2015; Mihalitsis and Bellwood 2017, 2019b; Carr and Motta 2020; Cohen, Weller, and Summers 2020; Cohen et al. 2020). Observations on feeding performance in piscivorous fishes routinely suggest a period of prey manipulation following capture (Reimchen 1991; Juanes and Conover 1994; Grubich et al. 2008). However, such behaviors involved in the processing of prey are rarely quantified. Unlike some mammals, fishes lack limbs that can be utilized to manipulate captured prey, and fishes may have to rely on their oral teeth to process prey prior to ingestion (Dean et al. 2005; Kolmann et al. 2016). As the successful acquisition of food relies on both prey capture and processing, assessing the role of oral teeth with reference to both morphology and behavior is required to accurately describe the functional role of oral teeth within piscivorous fishes.

The field of forensic odontology (Avon 2004; Saxena et al. 2010), although primarily focused on human teeth, can be extended to include analysis of animal bite marks/wounds to provide information on the identity of the perpetrator and how the teeth were utilized during the attack (Murmann et al. 2006; Bury et al. 2012; de Siqueira et al. 2016; Ressel et al. 2016). The underlying assumption within these studies is that tooth marks provide direct evidence linking tooth morphology and function. For example, in sharks, bite marks and the nature of resultant wounds have been used to determine both the size and species of attacking predators (Lowry et al. 2009; Jublier and Clua 2018), as well as the strike behavior of individuals (Ritter and Levine 2004, 2005). In a similar manner, bite wounds on prey fish may serve as a direct indicator of how the teeth of piscivorous fishes are used in both the capturing and processing of prey.

As an extension to forensic odontology, we develop a comparative framework to directly relate the resultant bite marks and wound patterns on prey fishes to the morphology and behavior of piscivorous fishes. This was carried out by comparing feeding-induced damage inflicted by two functionally distinct piscivore functional groups—grabbers and engulfers—to determine the function of oral dentitions in piscivorous fishes. Utilizing this framework to delineate functional relationships in feeding within piscivorous fishes, we address existing knowledge gaps by moving beyond inferring function from morphology and/or behavior in isolation. We also provide empirical evidence, revealing how specific traits are utilized, in concert, during both prey capture and processing. Findings from this study may thus provide additional insights into the mechanisms underpinning disparate feeding strategies in piscivorous reef fishes.

## Materials and methods

### Performance experiments

Aquarium-based experiments were conducted between 2018 and 2021 at James Cook University (JCU), Townsville, Australia. Experimental design and fish husbandry were conducted according to the guidelines stipulated under the JCU Animal Welfare and Ethics Committee (A2523). A total of 12 species of fishes were included in this study (see Supplemental data, Fig. S1). Depending on availability, 1–3 sub-adults/adults from each species were acquired from commercial aquarium suppliers. Following Mihalitsis & Bellwood (2021), piscivores were categorized into the following functional groups: grabbers (possessing macrodont dentition) and engulfers (possessing edentulate and villiform dentition). While some piscivores may have small, dentigerous (teeth-bearing) bones within their oropharyngeal cavity (e.g., on the vomer and palatine), the morphology of these tooth patches (small and villiform), and their location within the oropharyngeal cavity, suggest that these teeth are unlikely to cause any significant damage (Mihalitsis and Bellwood 2019b, 2021). We therefore excluded these teeth from subsequent morphological measurements. Additionally, within our two functional groups—grabbers and engulfers—we noted those species possessing modified pharyngeal jaws (with teeth), categorizing them as either grabber-pharyngognath or engulfer-pharyngognath (for allocations, see Supplemental data, Fig. S1). Although pharyngognathy is a widely recognized evolutionary modification (Wainwright et al. 2012; Burress 2015), it is used for processing food after capture, and hence may not be related to the capturing behavior of these fishes. Pharyngognathy was, thus, not the primary focus of this study.

Piscivores were housed individually in 20-L or 120-L tanks (in a climate-controlled room at 27°C) with a filtered flow-through water system. 20-L tanks were chosen for small, ‘‘sit-and-wait’’ ambush predators, while 120-L tanks were used for larger, ‘‘active’’ predators. Halogen lights above the tanks were switched on between 08:00 and 18:00 h. Piscivores were held for at least 1 week before commencing performance experiments and were fed commercially available food (chopped prawn) to acclimatize them to the experimental setup and the subsequent performance experiment. *Acanthochromis polyacanthus* (f. Pomacentridae) is known to be both abundant and common prey for piscivores on the Great Barrier Reef (Graham et al. 2003), and was thus used as prey in all performance experiments. These fishes were housed in separate large holding tanks and were fed daily with commercial flake food.

To account for the influence of satiation on piscivore performance, piscivores were starved for 24 h prior to experiments. To document the feeding event, a Go-Pro Hero 4 camera and a Sony RX100 IV camera were used to obtain real-time and slow-motion footage for subsequent video analysis. These were positioned in front of the experimental tanks. Prior to each feeding event, measurements of the standard length (SL) and maximum depth (MD) of prey fish were taken. The MD was measured from the highest point on the body to the gap between the pelvic and anal fins (using vernier calipers to the nearest 0.1 mm). Measurements were taken while holding the prey in a zip-lock bag to avoid influencing any olfactory or chemical cues. The sizes of prey used in feeding experiments were consistently selected to be more than 50% of the horizontal oral gape of the individual piscivore so that these predators could perform close to their maximal abilities (cf. Wainwright and Reilly 1994; Mihalitsis and Bellwood 2017).

A solid white opaque partition was used to divide the tank, with the piscivore restricted to one side. A single prey fish was introduced into the uninhabited space and was allowed to orient for 1 min. The partition was then removed, and the subsequent feeding event was filmed for the duration of the event. If the piscivore failed to strike the prey fish within 1 min, the prey was removed. After the piscivore had fully ingested the prey fish, the piscivore was removed from the tank and euthanized using clove oil followed by an ice-slurry mixture (A2523). Morphological measurements and photos of both predator and prey were taken within the next 20 min (see Supplemental data, Table S1). Using a Nikon D200 camera, photos of piscivores (displayed laterally) were taken from above with the camera oriented perpendicular to the fish, with a pin (head diameter = 5 mm) positioned within the image for scaling purposes in subsequent image analyses. The SL was measured to the nearest 0.1 mm and the mass to the nearest 0.001 g. The horizontal oral gape was measured using a pair of dissection scissors (following Mihalitsis and Bellwood 2017). Subsequently, the adductor mandibulae (AM) complex (consisting of the A1, A2, and A3 subdivisions) was removed and weighed to the nearest 0.001 g. For premaxillary jaw protrusion and the length of the longest tooth on the lower oral jaw, measurements were carried out using the line tool in ImageJ (Schneider et al. 2012) using the pin scale as a reference. Protrusion was recorded by calculating the difference between the anterior-most tip of the eye and the tip of the upper jaw when the mouth was open and closed, while photographs of piscivores with open mouths were used to measure the longest tooth on the lower jaw. Morphological measurements of each piscivorous fish were standardized to body size to permit comparisons of traits among species (see Supplemental data, Table S1). Similarly, relative AM weight was standardized to the piscivore's mass. Meanwhile, relative prey size was calculated as a proportion of the prey's MD to the piscivore's horizontal oral gape. To test for allometric relationships, body-standardized traits were plotted against body size. As no significant relationships were found, these body-standardized traits were used in subsequent analyses.

The mode of prey-capture by the piscivore was quantified from videos of performance experiments. This was done by observing the location of the initial strike by the piscivore on the prey and assigning it to one of three categories: head-first, mid-body-first, or tail-first. The prey position when swallowed was also noted as either head-first or tail-first. Additionally, the number of predator headshakes and bites were recorded for each piscivore. A headshake was defined as a vigorous, rapid, lateral head movement (lasting ∼ 0.5–2 s) following prey capture (occasionally the prey were hit against the aquarium wall/bottom). Bites were defined as the number of times that the predator utilized its oral teeth (opening and closing of oral jaws) on the prey following the initial first bite used to capture the prey.

### Quantification of damage to prey

**Fig. 1:**
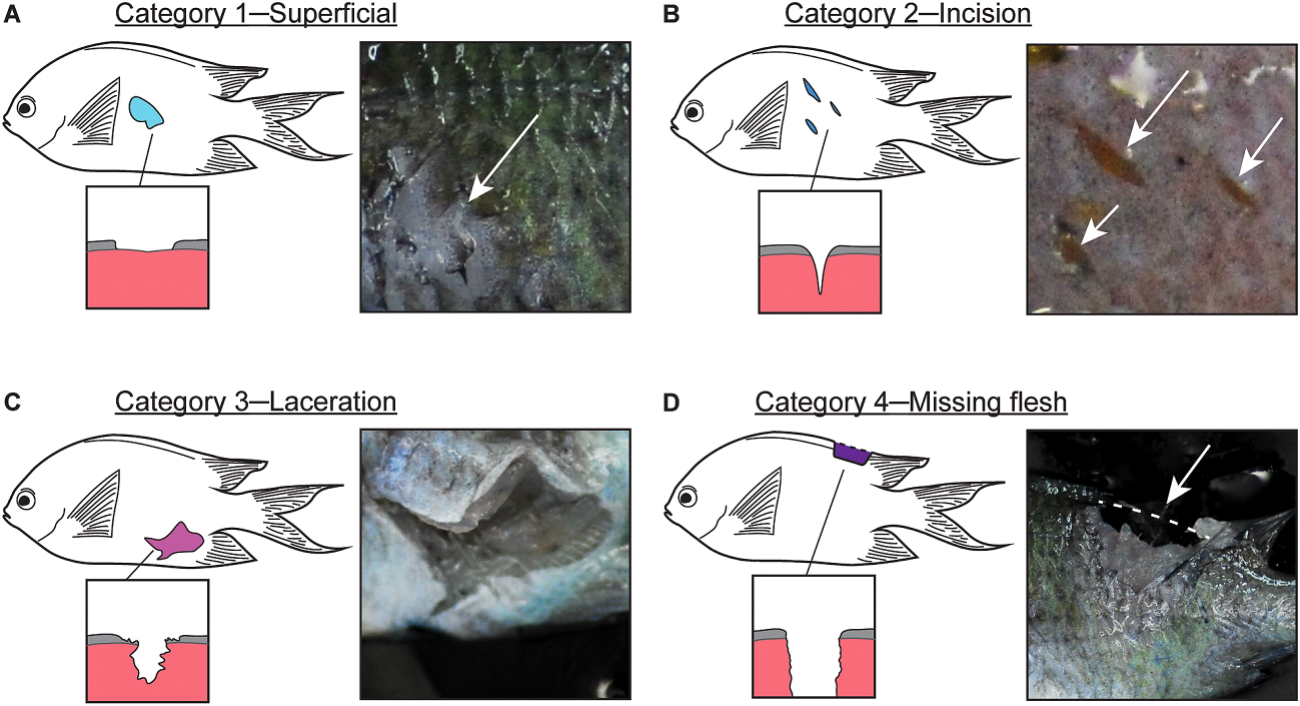
Damage categories observed on prey fish with respective colors assigned: (**A**) category 1––superficial (cyan), (**B**) category 2––incision (blue), (**C**) category 3––laceration (purple), (**D**) category 4––missing flesh (dark purple). Boxes display the margins and perceived depth (not to scale) of the wounds (left) and the actual wounds (right).

Following morphological measurements of piscivores, the prey was removed from the piscivore's stomach. This was carried out by carefully creating an incision on the lower abdomen of the piscivore, followed by a lateral incision in the stomach of the piscivore. The prey fish was then carefully removed using a pair of forceps. This was performed slowly and methodically to prevent any further damage to the prey during removal. Once removed, the left and right sides of the prey fish were photographed by laying the prey fish laterally with a scale positioned within the image. ImageJ was then used to quantify the extent of the damage inflicted on each prey fish. This was carried out by using the polygon tool to outline and calculate the area of wounds. Following visual examination of wounds, these damage areas were then assigned to one of four damage categories: category 1—superficial, category 2—incision, category 3—laceration, and category 4—missing flesh. Categories were defined in increasing order of severity following forensic veterinary pathological studies (de Siqueira et al. 2016; Ressel et al. 2016). Categories were based on the margins surrounding the wound, the perceived depth of wound and the damage (or lack thereof) to tissues in and around the wound (Fig. 1). Given the lack of studies that have explicitly investigated wounds in fishes, we categorized wounds solely based on their morphology.

Category 1 (superficial) damage was defined as any damage to the scales or integument of the prey that did not penetrate the underlying tissues (Fig. 1A). Category 2 (incision) damage was defined as a wound that exhibited damage to both the integument and underlying tissue. Incised wounds had regular and distinct margins (usually sharp or angular) so that the normal structure of the tissue surrounding the wound was not altered (de Siqueira et al. 2016; Ressel et al. 2016) (Fig. 1B). Category 3 (laceration) damage was defined as a tear in both the integument and the underlying tissue. However, it differed from category 2 (incision) in that these wounds displayed considerable damage to the natural structure of the tissue surrounding the wound. Lacerations were characterized by irregularly shaped wounds with ragged margins (Fig. 1C) (de Siqueira et al. 2016; Ressel et al. 2016). Lastly, category 4 (missing flesh) damage was assigned to areas where tissue had been removed from the natural outline of the prey fish (Fig. 1D). For this category, the outline of the prey was compared to an intact *A. polyacanthus*. The relative extent of the damage in each category was calculated as the proportion of the area of each damage category relative to the total area of the prey fish. Fins were excluded from this total area. Relative total damage was then calculated as the sum of all four relative damage categories. Given that each prey fish yielded a left and right image, all calculations were taken as an average of the left and right side of each fish.

### Heatmap plotting

To visualize the location of damage inflicted on prey, heatmaps for each functional group were plotted using the *patternize* package in R Studio version 1.2.5042 (R Core Team 2020) using the methods described in Van Belleghem et al. (2018) and Hemingson et al. (2019). To do this, damage areas within each prey photograph were first colored according to their respective damage categories (Fig. 1) using ImageJ. Images displaying the right side of prey fish were flipped such that all photographs were oriented in the same manner (anterior of fish facing left). Using the multi-point tool, 24 landmarks were placed at fixed morphological positions on each photograph (both left and right side) of the prey fish. Within functional groups, these images were then aligned and transformed to a target image using the landmarks as reference (Van Belleghem et al. 2018). This landmark-based transformation accounted for non-uniform changes in shape between different sized prey and the target image by carrying out a thin plate spline transformation (Duchon 1977), based on the 24 landmarks within each photo. During landmark-based transformations, the damage areas were extracted by denoting the red, green, blue (RGB) values of the specific color used to fill the damage polygons, allowing damage areas belonging to each category to be extracted. This process produced a raster image containing the sum of extracted damage areas, which was then plotted on the target image of a prey fish to serve as a background for visualization. The final output yielded heatmaps that indicate the probability of observing any single damage category at specific locations on a prey fish.

### Statistical analyses

All statistical analyses were conducted in R Studio. Given that species are non-independent due to shared ancestry (Felsenstein 1985), phylogenetic relationships between piscivorous species were considered in the analyses. To quantify this, a phylogenetic tree (see Supplemental data, Fig. S1) was constructed using the *rotl* package (Michonneau et al. 2016), based on the Open Tree of Life database (Open Tree of Life et al. 2019) with branch lengths computed based on the Grafen transformation (Grafen 1989). A phylogenetic principal component analysis (pPCA) with a Brownian motion correlation structure under the *phytools* package (Revell 2012) was then used to test for differences between the two functional groups (engulfer and grabber), based on functional traits and total damage. For piscivore species with two or three individuals, an average of the aforementioned variables was obtained. Additionally, to compare how inflicted damage varied between pharyngognath and non-pharyngognath piscivores, a non-parametric Kruskal–Wallis test was used.

To explore the relationships between the morphological and behavioral traits (for traits tested see Supplemental data, Table S2) and each of the damage categories, phylogenetic generalized least squares (PGLS) analyses were conducted using the *nlme* package (Pinheiro et al. 2021). Pharyngognath morphotypes were excluded from these analyses as damage likely included processing by the pharyngeal teeth, which were not the primary focus of this study. PGLS models were conducted using a Brownian motion correlation structure and a maximum likelihood estimation. To determine the model of best fit, models were compared according to the second-order Akaike information criterion (AICc).

## Results

### Engulfers versus grabbers

**Fig. 2:**
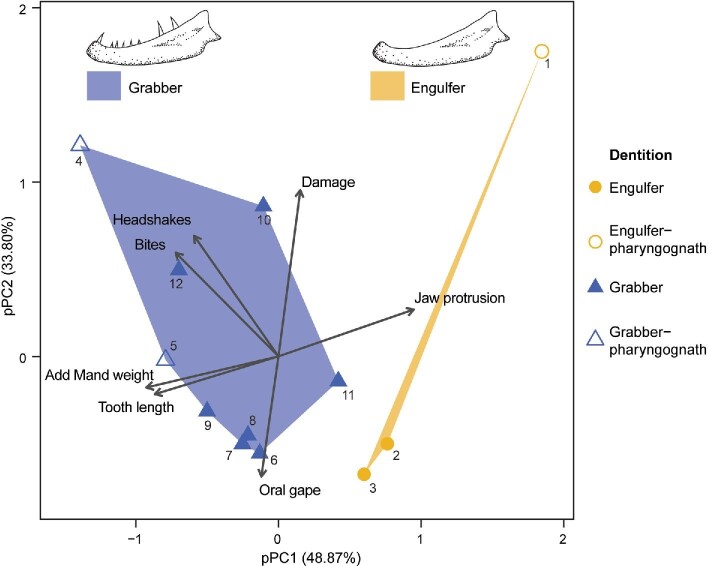
The pPCA was based on morphological and behavioral traits as well as total relative prey damage, in 12 species of piscivorous fishes, which were categorized according to their dentition. Blue and yellow polygons are used to denote grabber and engulfer species, respectively. Species: (1) *Epibulus insidiator*, (2) *Dendrochirus zebra*, (3) *Pterois volitans*, (4) *Oxycheilinus digramma*, (5) *Oxycheilinus unifasciatus*, (6) *Cheilodipterus quinquelineatus*, (7) *Lutjanus argentimaculatus*, (8) *Lutjanus bohar*, (9) *Lutjanus russellii*, (10) *Ogilbyina queenslandiae*, (11) *Pseudochromis fuscus*, and (12) *Paracirrhites forsteri*.

The pPCA explained 48.9% (pPC1) and 33.8% (pPC2) of the total variation (Fig. 2). The separation of engulfers and grabbers primarily along pPC1 supports the distinction of these two piscivorous functional groups (Fig. 2). Engulfers possess relatively greater jaw protrusion and relatively smaller adductor mandibulae (AM) complexes compared to grabbers. In contrast, grabbers exhibit relatively less jaw protrusion and relatively larger AM complexes (Fig. 2).

**Fig. 3:**
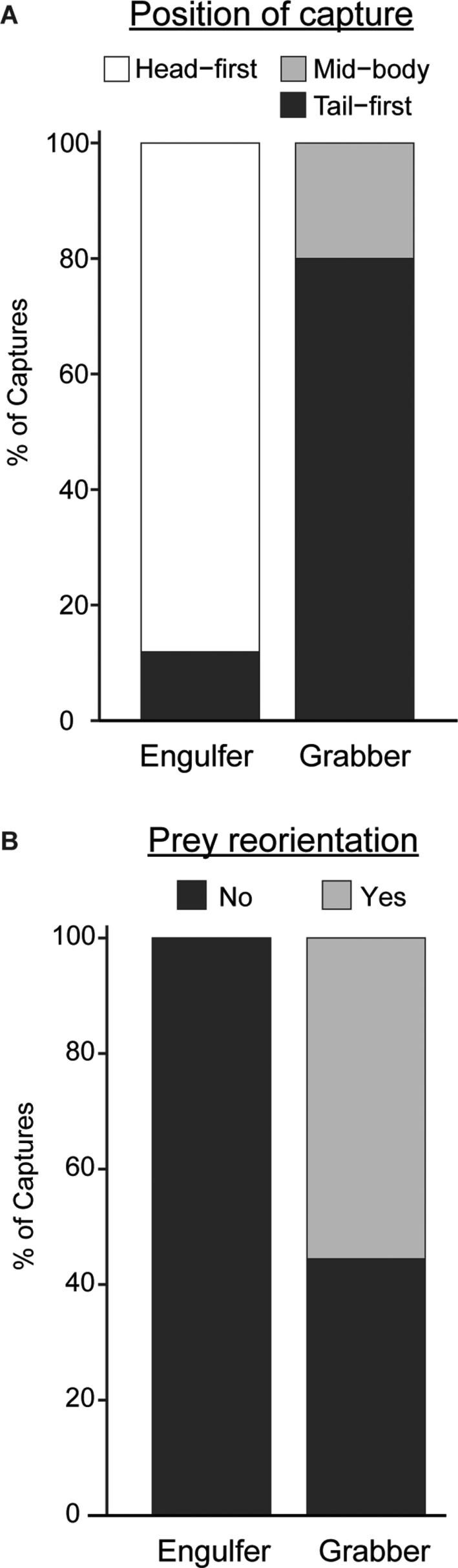
(**A**) Orientation of prey as a proportion of prey capture events for engulfers and grabbers functional groups. (**B**) Frequency of prey reorientation (i.e., bite, spit, and recaptured head-first).

**Fig. 4:**
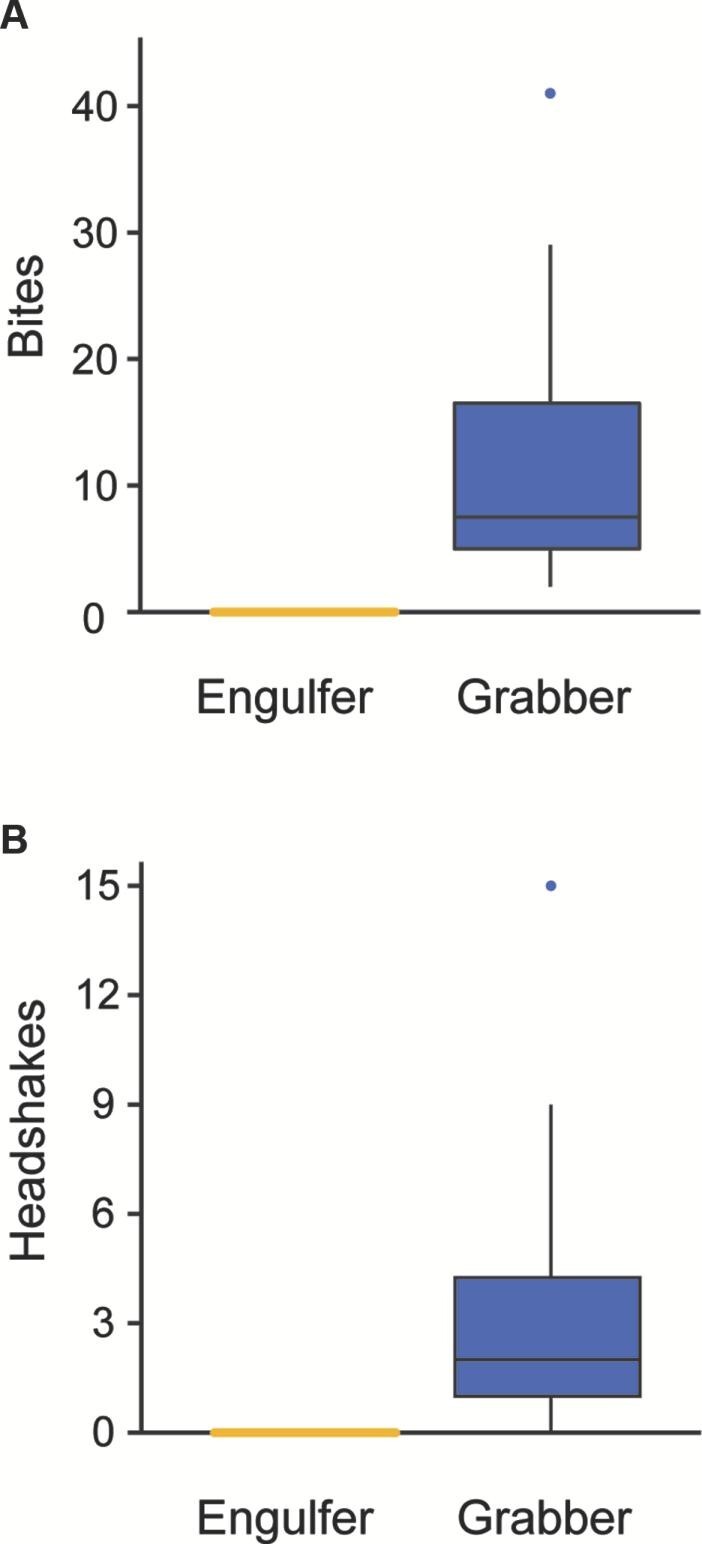
Boxplots showing the number of bites (**A**) and headshakes (**B**) following the initial capture of prey for both engulfers (yellow) and grabbers (blue). Median, quantiles, minimum/maximum values (whiskers), and outliers are denoted. Note that no bites or headshakes were recorded for engulfers.

During performance experiments, head-first capturing and subsequent swallowing of prey was observed in 87.5% of engulfers (*n* = 8) (Fig. 3). No bites or headshakes were recorded for engulfers (Fig. 4). For grabbers (*n* = 10), tail-first capture was observed 80% of the time, with the other 20% being mid-body captures (Fig. 3). Following capture, bites were always observed (100%) in grabbers, while headshakes occurred in 80% of grabber feeding events (Fig. 4). Essentially, engulfers were observed to capture and ingest their prey head-first, whereas grabbers captured prey tail-first and processed them through multiple bites and headshakes. Furthermore, 55.6% of grabbers were subsequently observed spitting prey out and then recapturing them head-first for subsequent ingestion (Fig. 3).

### Prey damage—non-pharyngognaths

When quantifying total damage inflicted on prey fish by non-pharyngognath piscivores, prey from grabbers had significantly greater total damage areas compared to prey from engulfers (Kruskal–Wallis test; *H*_1_ = 6.19, *P* < 0.05). Prey from grabbers recorded a mean total damage of 11.1% (standard error [SE] ± 3.6%) of their body area, while prey from engulfers recorded a mean total damage of just 0.8% (SE ± 0.5%) of their body area.

**Fig. 5:**
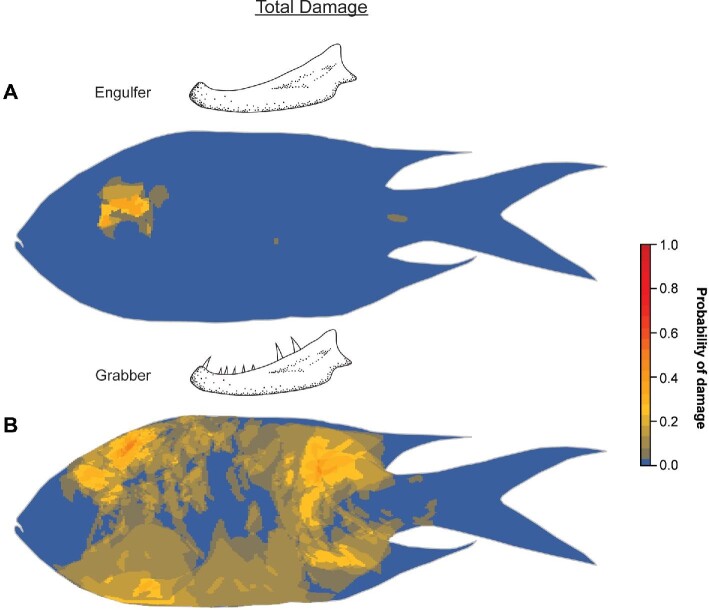
Heatmaps showing the probability of occurrence of total damage in any specific area on prey fish for (**A**) engulfers and (**B**) grabbers.

Looking at the location of damage on prey fish, heatmaps indicate that the area of total damage inflicted on prey is more widespread in grabbers compared to engulfers (Fig. 5). Among prey from grabbers, damage probability of approximately 60% was observed dorsal to the eye (approximate to the MD of the prey) and on the posterior end of the body, specifically, below the rear portion of the dorsal fin (Fig. 5B). Meanwhile, among prey from engulfers, damage areas were only observed immediately posterior to the eye, with less than a 40% probability of occurring (Fig. 5A).

**Fig. 6:**
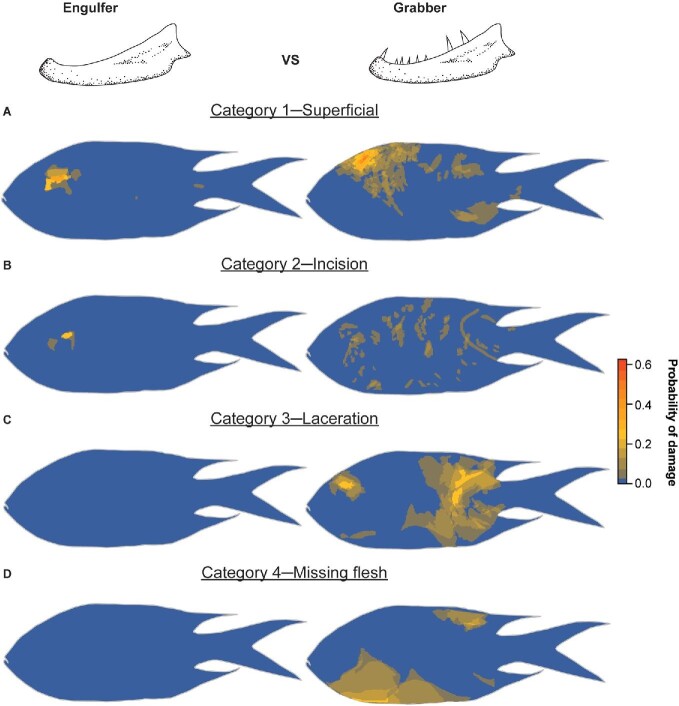
Heatmaps showing the probability of the occurrence of four damage categories at any specific area on prey fish in non-pharyngognath engulfers and grabbers. (**A**) Superficial, (**B**) incision, (**C**) laceration, and (**D**) missing flesh.

When looking at specific damage categories, prey from engulfers only recorded categories 1 and 2 damage, while prey from grabbers recorded all four damage categories (Fig. 6). Among prey from grabbers, category 1 (superficial) damage appears to be concentrated at the region posterior to the eye (Fig. 6A). Category 2 (incision) damage appears to occur across the entire body, with no clustering of damage in any region (Fig. 6B). In contrast, category 3 (laceration) damage appears to occur predominantly along the caudal (tail-end) of the prey (Fig. 6C). Meanwhile, category 4 (missing flesh) damage occurs primarily on the ventral region of the prey, coinciding with the softest part of the prey body (Fig. 6D).

**Fig. 7:**
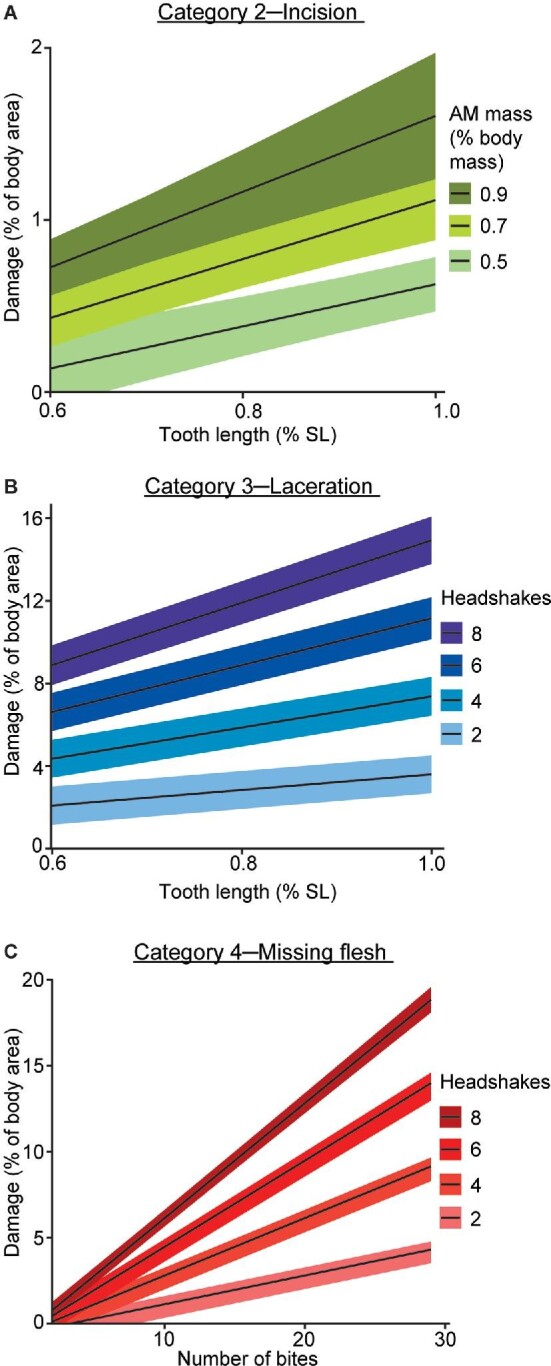
The PGLS models for (**A**) category 2––incision, (**B**) category 3––laceration, and (**C**) category 4––missing flesh damages. Interaction between two traits was found to be significant (*P* < 0.05) in each of the models: (A) relative tooth length (taken as % of the piscivore's SL) and relative AM mass (taken as % of the piscivore's mass), (B) relative tooth length and number of headshakes, and (C) number of bites and headshakes.

As engulfers recorded damage that was close to 0% of the total body area, we only conducted PGLS analyses (comparing traits and damage areas) for grabbers. When assessing each of the four damage categories inflicted on prey, the most parsimonious PGLS models (based on the AICc criteria; see Supplemental data, Table S2) for category 1 (superficial) damage were observed to be the null model (no traits included). This suggests that none of the traits measured best explained the extent of category 1 damage on prey. Meanwhile, for category 2 (incision) damage, the best model contained an interaction between relative tooth length and relative AM mass (*P* < 0.05, Fig. 7A), while for category 3 (laceration) damage, the best model contained an interaction between relative tooth length and the number of headshakes (*P* < 0.001, Fig. 7B). For category 4 (missing flesh) damage, the best model contained an interaction between the number of bites and the number of headshakes (*P* < 0.001, Fig. 7C). These results suggest that, firstly, a combination of the length of the longest tooth on the lowest jaw and AM mass was significant in explaining category 2 (incision) damage in prey fish. Meanwhile, a combination of the length of the longest tooth on the lowest jaw and the number of headshakes was significant in explaining category 3 (laceration) damage in prey fish, while a combination of bites and headshakes was significant in explaining the extent of category 4 (missing flesh) damage in prey fish. For both categories 3 and 4 damage, as the number of headshakes increases, the extent of damage observed on prey also increases (Fig. 7).

### Prey damage—pharyngognaths

Compared to non-pharyngognaths, pharyngognaths recorded greater mean relative total damage to prey (Kruskal–Wallis test; *H*_1_ = 10.274, *P* < 0.01). Prey from the engulfer-pharyngognath had on average, damage to 76.6% (SE ± 23.4%) of their body area, while prey from grabber-pharyngognaths had on average damage to 43.3% (SE ± 12.6%) of their body area. However, of the four damage categories, only category 3 (laceration) damage was found to be significantly greater in prey of pharyngognaths compared to non-pharyngognaths (Kruskal–Wallis test; *H*_1_ = 9.008, *P* < 0.01).

**Fig. 8:**
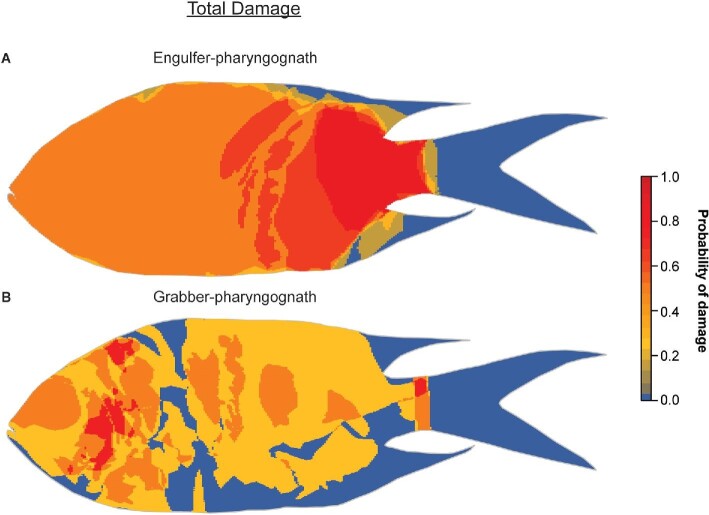
Heatmaps showing the probability of the occurrence of total damage at any specific area on prey fish for (**A**) an engulfer-pharyngognath and (**B**) grabber-pharyngognaths.

In comparing the location of total damage on prey fish, pharyngognaths were found to inflict extensive damage to prey, with multiple regions having more than a 60% probability of damage (Fig. 8). This damage predominantly consisted of category 3 (laceration) damage (see Supplemental data, Fig. S2C). Comparing both the extent and location of damage, the results suggest that pharyngognaths inflict a more severe form of damage to prey during processing.

## Discussion

By adopting a forensic odontological approach to assess morphological and behavioral traits in relation to bite damage in prey fish, our results confirm that engulfers and grabbers display fundamental differences in how they capture and process their prey. Engulfers are seen to capture and swallow their prey head-first, with no prey processing involved. In contrast, grabbers capture their prey predominantly tail-first and subsequently process their prey using their oral jaw teeth. Prey processing was observed in the form of headshakes and bites; behaviors that inflict severe damage to prey fish and, presumably, prevent escape. Additionally, within pharyngognath morphotypes, we provide evidence for the use of the pharyngeal jaws in processing prey by inflicting extensive damage during ingestion. These results highlight the importance of synchronously assessing morphological traits alongside behavior to delineate key functional traits. Specifically, the combination of morphological and behavioral attributes in this study reveals how piscivorous fishes use their oral jaw teeth during both prey capture and processing.

### Prey capture

In this study, engulfers capture their prey head-first, while grabbers predominantly capture prey tail-first. This difference in the mode of capture is consistent with previous studies examining feeding performances of species belonging to either of the aforementioned functional groups (Juanes and Conover 1994; Albins and Lyons 2012; Mihalitsis and Bellwood 2017, 2021). Given that a higher probability of prey escape has been associated with a tail-first capture compared to head-first (Reimchen 1991; Juanes and Conover 1994), grabbers likely rely on their teeth to grip and retain prey following capture (Nanami and Shimose 2013; Galloway et al. 2016). Within fishes, the AM complex contains muscles responsible for jaw adduction (Wainwright and Richard 1995; Westneat 2003). Compared to engulfers, grabbers have relatively larger AM complexes (Mihalitsis and Bellwood 2021), relying on a powerful or fast adduction of their jaws to bite firmly onto or into prey, to prevent escape (Huber et al. 2006; Habegger et al. 2011; Ferguson et al. 2015). Within engulfers, the lack of functional teeth to grip prey during capture (Mihalitsis and Bellwood 2019b) suggests that their AM complexes may have been freed from the role of providing powerful adduction during jaw closure. Instead, these fishes may rely more on their hypaxial and epaxial muscles to drive mouth expansion during feeding to engulf prey through a combination of ram and suction (Camp and Brainerd 2014; Camp 2019).

To capture prey, engulfers likely rely on both body ram (forward movement of the predator's body) and jaw ram (forward movement of the predator's jaw relative to its body) to lunge toward their prey (Longo et al. 2016). Jaw ram is facilitated by greater jaw protrusion, which was found to be higher in engulfers (Mihalitsis and Bellwood 2021). Jaw protrusion not only increases suction flow on prey (Holzman et al. 2008; Day et al. 2015), but also increases the speed at which prey is captured (Motta 1984; Oufiero et al. 2012; Wainwright et al. 2015). Thus, having greater jaw protrusion likely circumvents the need for engulfers to have functional teeth to grip and retain prey.

### Prey processing

Following head-first capture of prey in engulfers, prey processing was not observed for engulfers that ‘‘engulf”’ their prey whole and head-first. A preference for head-first ingestion of prey by piscivorous fishes has been recorded in several studies (Hoyle and Keast 1988; Mihalitsis and Bellwood 2017; Burke and Williamson 2021). Given that appendages on prey (spines, fin rays, scales, and opercula) tend to lay flat when prey is oriented head-first during ingestion, oesophageal abrasion arising from these structures is reduced during swallowing (Reimchen 1991). With prey engulfed whole and already oriented head-first during capture, engulfers likely do not need oral teeth to manipulate their prey into the desired orientation for transport further into the buccal cavity, therefore minimizing post-capture processing of prey. Given the lack of specialized pharyngeal jaws within non-pharyngognath piscivores, prey is likely transported further into the oesophagus (swallowing) by utilizing structures within the branchial basket (Weller et al. 2020). The small degree of damage behind the eye, the widest part of a fish, may reflect some degree of abrasion by small teeth in the buccal cavity or on branchial arches.

Grabbers in this study seemed to conform to a general sequence of behaviors that characterize a different type of prey processing. Following tail-first capture, grabbers repeatedly bite the prey, with intermittent headshakes, before either (1) swallowing the prey tail-first or (2) spitting the prey out and reorienting themselves to capture the prey head-first. Several studies have noted that toothed piscivores that captured prey tail-first (or mid-body), routinely spat out their prey and reoriented themselves to capture and ingest their prey head-first (Hoyle and Keast 1988; Reimchen 1991; Gill and Hart 1994; Mihalitsis and Bellwood 2017, 2021). This ‘‘bite, spit, and recapture’’ strategy, as documented herein, has also been observed in sharks (Motta 2004; Martin et al. 2005), which are known to bite firmly onto prey, drag them deeper within the water column and remove chunks of flesh (often the limbs) in the process. This damage impairs the movement of the prey, reduces the probability of escape, and may cause the prey to bleed out (Klimley 1994; Martin et al. 2005). In a similar manner, grabbing teleosts would need to be able to inflict sufficient damage during prey processing to incapacitate prey fish before spitting them out.

When assessing the type of damage inflicted on prey by grabbers in this study, our results highlight how morphological and behavioral traits work in unison to inflict the different categories of damage. Teeth, in conjunction with the larger AM muscles present in grabbers, were seen to inflict category 2 (incision) damage (Fig. 7A). Meanwhile, headshakes, in conjunction with teeth, were seen to inflict category 3 (laceration) and category 4 (missing flesh) damage (Fig. 7B and C). Incision wounds, which take on a more precise and surgical appearance, are likely the result of sharp teeth puncturing into the bodies of prey fish. Such a function requires a relatively large distance between caniniform teeth, or differences in the relative size between these teeth (Mihalitsis and Bellwood 2019b). The presence of large AM muscles (as mentioned earlier), combined with the dentition of a grabber (macrodont), is used to grip onto prey during the initial bite/capture. Once prey is captured, headshakes (with prey grasped between the teeth) allow grabbers to inflict more severe damage categories (laceration and missing flesh), which probably function to incapacitate prey and prevent escape. Doing so enables grabbers to spit out their prey, reorient themselves, and subsequently recapture and swallow the prey head-first.

Several studies have investigated the function of headshakes during prey processing. For example, observations on aquatic tetrapods, such as seals (Jones et al. 2013; Hocking et al. 2016), have suggested that headshaking movements following prey capture serve as a substitute to “hold and tear” prey processing, since the adapted forelimbs of these tetrapods have been rendered ineffective for grasping prey (Taylor 1987). This function of prey reduction by means of lateral headshaking has also been noted in several species of sharks (Motta 2004; Martin et al. 2005; Brewster et al. 2018) and the great barracuda, *Sphyraena barracuda* (Grubich et al. 2008; Habegger et al. 2011), as a means of cutting prey into smaller, manageable pieces for ingestion. This proposed function appears to be facilitated by the laterally flattened and/or serrated blade-like oral teeth in several species of sharks, which increase the shearing function of teeth from unidirectional movement during headshaking (Frazzetta and Prange 1987; Motta 2004; Huber et al. 2006; Whitenack and Motta 2010). In this study, prey of grabbers that displayed headshakes were not observed to be severed or cut into smaller pieces (as seen with sharks and the great barracuda). Headshakes may therefore take on another functional role during prey processing in piscivorous fishes with rounded caniniform teeth.

**Fig. 9:**
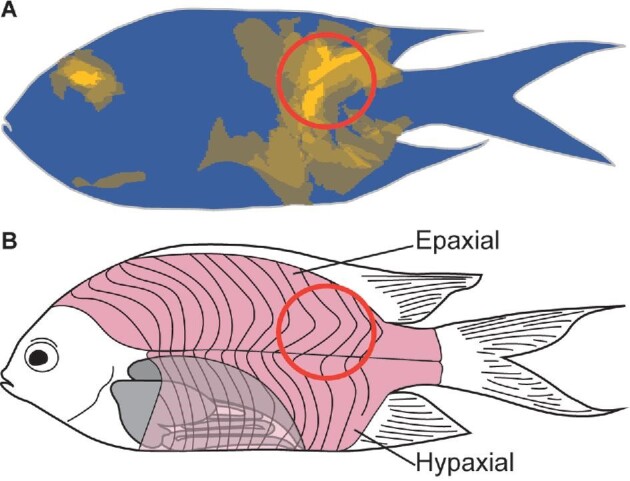
(**A**) Heatmap of grabber prey fish showing category 3––laceration damage. The red circle denotes the area where a high probability of laceration damage during prey capture and processing was observed, and (**B**) the corresponding locations on prey musculature.

Prey of grabbers displayed a high proportion of category 3 (laceration) damage in the epaxial musculature, particularly in the region close to the caudal fin (Fig. 9). Given the strong correlation between headshakes and category 3 damage (Fig. 7B), it is likely this behavior serves as a method of inflicting damage to the musculature of the prey by lateral movement of the teeth following a tail-first capture. To escape, fishes rely on the simultaneous activation of all regions of the epaxial musculature to carry out a fast-start escape response and reorient themselves away from danger (Jayne and Lauder 1995; Jimenez and Brainerd 2020). During this process, fishes undergo a bend of the body along the center of mass and subsequently rely on thrust generated by their caudal fin to swim away rapidly (Domenici and Blake 1997; Brainerd and Patek 1998). Damage to the epaxial musculature, particularly at the caudal region (Fig. 9), may severely impede the ability of prey fishes to swim away from a subsequent attack once spat out by the predator. Thus, conical caniniform teeth may serve several functions: first, to capture and retain prey during a tail-first capture by means of puncturing; and second, to inflict damage to key locomotive muscles in order to incapacitate prey and prevent escape when spat out. Grabbers can then recapture and swallow their prey head-first.

### Prey processing in pharyngognaths

The pharyngeal jaws of pharyngognath morphotypes enabled extensive prey processing by primarily inflicting category 3 (laceration) damage to large areas of the prey fish (see Supplemental data, Fig. S2C). This extensive damage is unlikely to be attributed to the oral jaws of grabber-pharyngognaths, as the engulfer-pharyngognath did not possess functional oral teeth and yet displayed similarly high category 3 damage to prey fish. Lacerating deep into prey may reduce the body thickness of larger prey by breaking and reducing external features (i.e., spines and bones) on prey fish. Swallowing prey requires the particular food item to fit within the pharyngeal gape of the piscivore (Mihalitsis and Bellwood 2017). Processing by means of laceration may reduce these limitations, especially considering the smaller pharyngeal gape that has been associated with pharyngognathy in piscivorous cichlids (McGee et al. 2015; Burress et al. 2016). The damage caused by pharyngeal jaws is not related to prey capturing, but prey processing. Damage caused by pharyngeal jaws in piscivorous fishes may therefore be linked to size reduction, or shape manipulation of the prey, enabling it to fit through the pharyngeal gape.

Interestingly, grabber-pharyngognaths in this study were observed to carry out headshakes once prey had been captured (tail-first), aligning within the ‘‘bite, spit, and recapture’’ strategy. Thus, headshakes may also be used by grabber-pharyngognaths to incapacitate prey and prevent escape, thereby facilitating a head-first ingestion once prey has been spat out. This independent use of headshakes in species with a well-developed processing apparatus emphasizes that headshakes are most likely used for prey incapacitation and not processing for subsequent ingestion or digestion.

## Conclusion

The two distinct functional groups of piscivorous fishes, engulfers and grabbers, were found to differ in their feeding strategies based on morphological and behavioral traits. Essentially, engulfers engulf prey whole and head-first with no oral teeth to grip and retain prey. In contrast, grabbers grab their prey tail-first and process their prey by means of headshakes and bites. Headshakes appear to be used primarily to incapacitate prey by lacerating the muscles responsible for prey escape, allowing grabbers to spit out, recapture, and swallow prey head-first. By adopting a novel forensic odontological approach to quantify and categorize bite wounds in prey fish, this study was able to describe the functional role of oral jaw teeth in piscivorous fishes during both prey capture and processing.

## Supplementary Material

obac011_Supplemental_Files
