# Supplementary material for: Forensic odontology: Assessing bite wounds to determine the role of teeth in piscivorous fishes
Source: Integr Org Biol. 2022 Mar 12;4(1):obac011. doi: 10.1093/iob/obac011 (PMC9053946; doi:10.1093/iob/obac011)
Supplement: obac011_Supplemental_Files [file obac011_supplemental_files.zip › Figure_Caption.docx]

**Fig. 1** Damage categories observed on prey fish with respective colours assigned: (**A**) Category 1─Superficial (cyan), (**B**) Category 2─Incision (blue), (**C**) Category 3─Laceration (purple), (**D**) Category 4─Missing flesh (dark purple). Boxes display the margins and perceived depth (not to scale) of the wounds (left) and the actual wounds (right).

**Fig. 2** Phylogenetic principal component analysis (PPCA) based on morphological and behavioural traits, as well as total relative prey damage, in 12 species of piscivorous fishes which were categorised according to their dentition. A blue and yellow polygon are used to denote grabber and engulfer species respectively. Species: 1─*Epibulus insidiator*, 2─*Dendrochirus zebra*, 3─*Pterois volitans*, 4─*Oxycheilinus digramma*, 5─*Oxycheilinus unifasciatus*, 6─*Cheilodipterus quinquelineatus*, 7─*Lutjanus argentimaculatus*, 8─*Lutjanus bohar*, 9─*Lutjanus russellii*, 10─*Ogilbyina queenslandiae*, 11─*Pseudochromis fuscus*, 12─*Paracirrhites forsteri*.

**Fig. 3** (A) Orientation of prey as a proportion of prey capture events for engulfer and grabber functional groups. (B) Frequency of prey reorientation (i.e. bite, spit and recaptured head-first).

**Fig. 4** Boxplots showing the number of bites (A) and headshakes (B) following the initial capture of prey for both engulfers (yellow) and grabbers (blue). Median, quantiles, minimum/maximum values (whiskers) and outliers are denoted. Note that no bites or headshakes were recorded for engulfers.

**Fig. 5** Heatmaps showing the probability of occurrence of total damage in any specific area on prey fish for (**A**) engulfers and (**B**) grabbers.

**Fig. 6** Heatmaps showing the probability of occurrence of four damage categories at any specific area on prey fish in non-pharyngognath engulfers and grabbers. (**A**) Superficial, (**B**) Incision (**C**) Laceration, and (**D**) Missing flesh.

**Fig. 7** Phylogenetic generalised least squares (PGLS) models for (**A**) Category 2─Incision, (**B**) Category 3─Laceration, (**C**) Category 4─Missing flesh damages. Interaction between two traits were found to be significant (p<0.05) in each of the models: (**A**) relative tooth length (taken as % of piscivore’s SL) and relative AM mass (taken as % of piscivore’s mass), (B) relative tooth length and number of headshakes, and (C) number of bites and headshakes.

**Fig. 8** Heatmaps showing the probability of occurrence of total damage at any specific area on prey fish for (**A**) an engulfer-pharyngognath and (**B**) grabber-pharyngognaths.

**Fig. 9** (**A**) Heatmap of grabber prey fish showing Category 3─Laceration damage. The red circle denotes the area where a high probability of laceration damage ­­during prey capture and processing was observed, and (**B**) the corresponding locations on prey musculature.
