# Supplementary material for: Forensic odontology: Assessing bite wounds to determine the role of teeth in piscivorous fishes
Source: Integr Org Biol. 2022 Mar 12;4(1):obac011. doi: 10.1093/iob/obac011 (PMC9053946; doi:10.1093/iob/obac011)
Supplement: obac011_Supplemental_Files [file obac011_supplemental_files.zip › Supplemental Material.docx]

**Supplemental information**

**
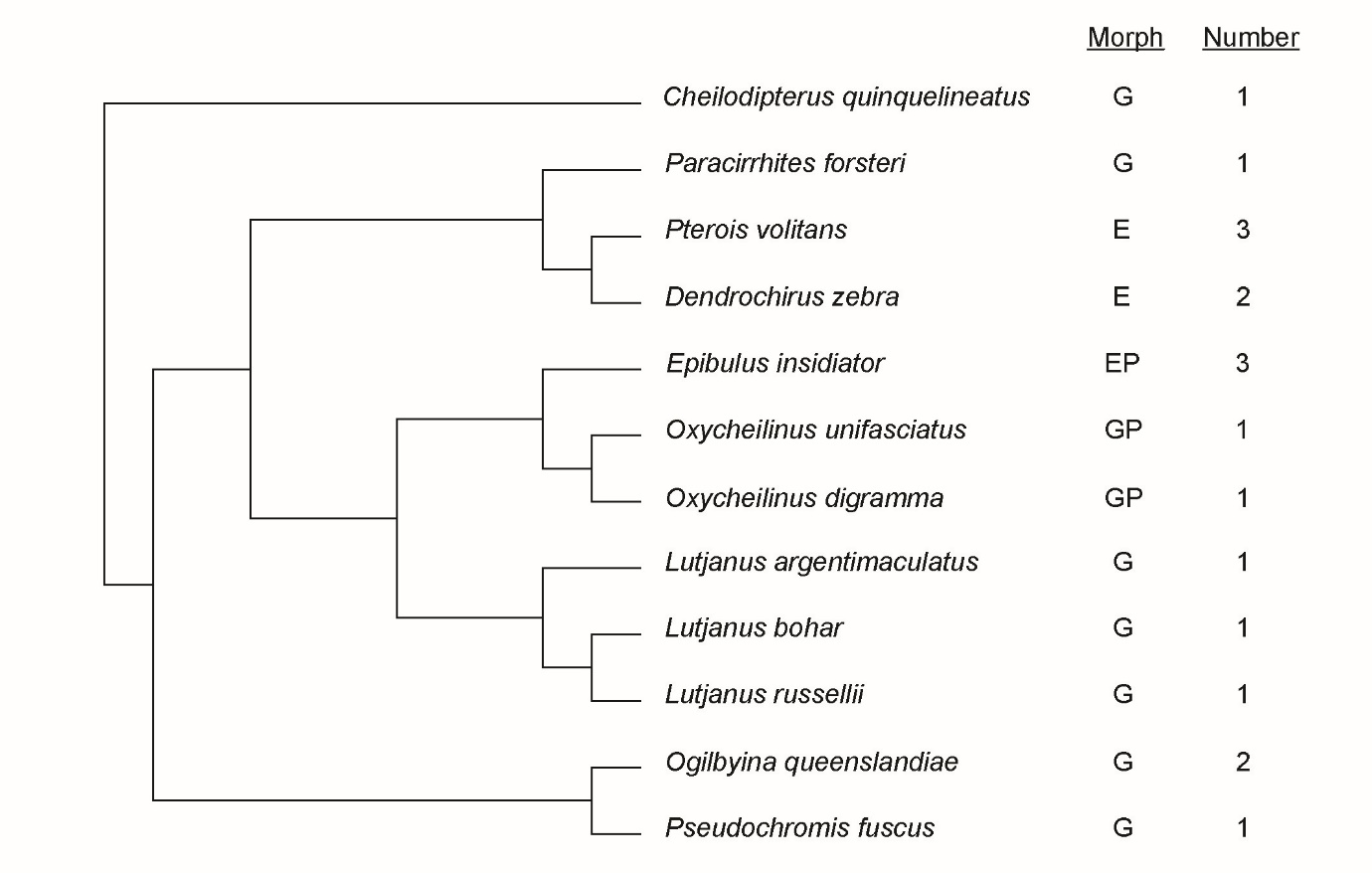
**

**Fig. S1.** Phylogenetic tree showing the 12 species of piscivorous fishes (and number of individuals) used in this study, separated by morphotype (G: Grabber, E: Engulfer, EP: Engulfer-pharyngognath, GP: Grabber-pharyngognath).

**Table S1** Trait and damage data in 12 species of piscivores (mean ± SE shown for species with n > 1) separated by predator group (EP: Engulfer-pharyngognath, E: Engulfer, GP: Grabber-pharyngognath, G: Grabber). (%) Values calculated as relative percentages where appropriate.

| Species | Morpho-type | n | Horizontal oral gape (%) | Tooth length (%) | Protrusion (%) | AM weight (%) | Bites | Head shakes |  | Total damage (%) | Superficial (%) | Incision (%) | Laceration (%) | Missing flesh (%) |
| --- | --- | --- | --- | --- | --- | --- | --- | --- | --- | --- | --- | --- | --- | --- |
| *Epibulus insidiator* | EP | 3 | 8.45 ±  0.40 | NA | 20.57 ± 1.42 | 0.32 ± 0.01 | 0.00 | 0.00 |  | 76.63 ± 23.37 | 0.00 ±  0.00 | 0.10 ± 0.10 | 66.56 ± 31.49 | 9.98 ± 8.09 |
| *Dendrochirus zebra* | E | 2 | 23.52 ± 1.48 | NA | 7.82 ± 0.79 | 0.47 ± 0.01 | 0.00 | 0.00 |  | 1.97 ± 0.77 | 1.71 ± 0.64 | 0.26 ± 0.13 | 0.00 ± 0.00 | 0.00 ± 0.00 |
| *Pterois volitans* | E | 3 | 25.51 ± 1.41 | NA | 6.09 ± 0.41 | 0.52 ± 0.04 | 0.00 | 0.00 |  | 0.01 ± 0.01 | 0.01 ± 0.01 | 0.00 ± 0.00 | 0.00 ± 0.00 | 0.00 ± 0.00 |
| *Oxycheilinus digramma* | GP | 1 | 16.48 | 1.48 | 2.41 | 0.95 | 41.00 | 9.00 |  | 55.90 | 1.99 | 1.60 | 39.66 | 12.65 |
| *Oxycheilinus unifasciatus* | GP | 1 | 15.59 | 1.70 | 3.35 | 0.96 | 5.00 | 0.00 |  | 30.78 | 1.84 | 1.39 | 27.54 | 0.00 |
| *Cheilodipterus quinquelineatus* | G | 1 | 25.25 | 1.00 | 1.72 | 0.51 | 2.00 | 2.00 |  | 12.07 | 6.72 | 0.35 | 5.00 | 0.00 |
| *Lutjanus argentimaculatus* | G | 1 | 21.29 | 0.71 | 3.49 | 0.80 | 5.00 | 1.00 |  | 4.81 | 3.58 | 1.24 | 0.00 | 0.00 |
| *Lutjanus bohar* | G | 1 | 21.90 | 0.64 | 4.58 | 0.77 | 8.00 | 2.00 |  | 2.55 | 1.52 | 1.03 | 0.00 | 0.00 |
| *Lutjanus russellii* | G | 1 | 17.23 | 0.584 | 2.16 | 0.95 | 12.00 | 1.00 |  | 1.71 | 0.59 | 1.12 | 0.00 | 0.00 |
| *Ogilbyina queenslandiae* | G | 2 | 10.56 ± 0.19 | 0.66 ± 0.15 | 2.54 ± 0.72 | 0.48 ± 0.06 | 12.50 ± 5.50 | 8.50 ± 6.50 |  | 22.93 ± 5.44 | 2.97 ± 0.14 | 0.26 ± 0.26 | 10.66 ± 2.60 | 9.05 ± 8.15 |
| *Pseudochromis fuscus* | G | 1 | 14.15 | 0.62 | 4.25 | 0.41 | 2.00 | 0.00 |  | 1.52 | 1.52 | 0.00 | 0.00 | 0.00 |
| *Paracirrhites forsteri* | G | 1 | 15.02 | 0.87 | 1.73 | 0.76 | 29.00 | 5.00 |  | 20.27 | 1.21 | 0.91 | 7.34 | 10.81 |

**Table S2** Phylogenetic generalised least squares (PGLS) model summary for each damage category for grabbers. Models displayed for each category are the ones with the lowest AICc.

| Category (Response) | Trait (Explanatory) | Observations | AICC | Log-Lik | Estimate | Std. Error | Conf. Int (95%) | t-value | p-value |
| --- | --- | --- | --- | --- | --- | --- | --- | --- | --- |
| **Total damage**  **(4+3+2+1)** | (Intercept) | 7 | 45.27 | -15.63 | 2.45 | 2.15 | -1.76 – 6.66 | 1.14 | 0.305 |
|  | Relative tooth length x Headshakes |  |  |  | 3.83 | 0.28 | 3.29 – 4.38 | 13.77 | **<0.001** |
| **Missing flesh**  **(Category 4)** | (Intercept) | 7 | 24.65 | -5.32 | -0.54 | 0.49 | -1.50 – 0.42 | -1.11 | 0.318 |
|  | Bites x Headshakes |  |  |  | 0.08 | 0.00 | 0.08 – 0.09 | 27.14 | **<0.001** |
| **Laceration**  **(Category 3)** | (Intercept) | 7 | 33.98 | -9.99 | -0.18 | 0.96 | -2.06 – 1.70 | -0.19 | 0.860 |
|  | Relative tooth length x Headshakes |  |  |  | 1.89 | 0.12 | 1.64 – 2.13 | 15.19 | **<0.001** |
| **Incision**  **(Category 2)** | (Intercept) | 7 | 9.404 | 2.29 | -0.60 | 0.42 | -1.43 – 0.23 | -1.41 | **0.217** |
|  | Relative AM mass x Relative tooth length |  |  |  | 2.45 | 0.81 | 0.87 – 4.03 | 3.03 | **0.029** |
| **Superficial**  **(Category 1)** | (Intercept) | 7 | 36.28 | -14.64 | 3.32 | 1.62 | 0.15 – 6.48 | 2.05 | 0.086 |

**
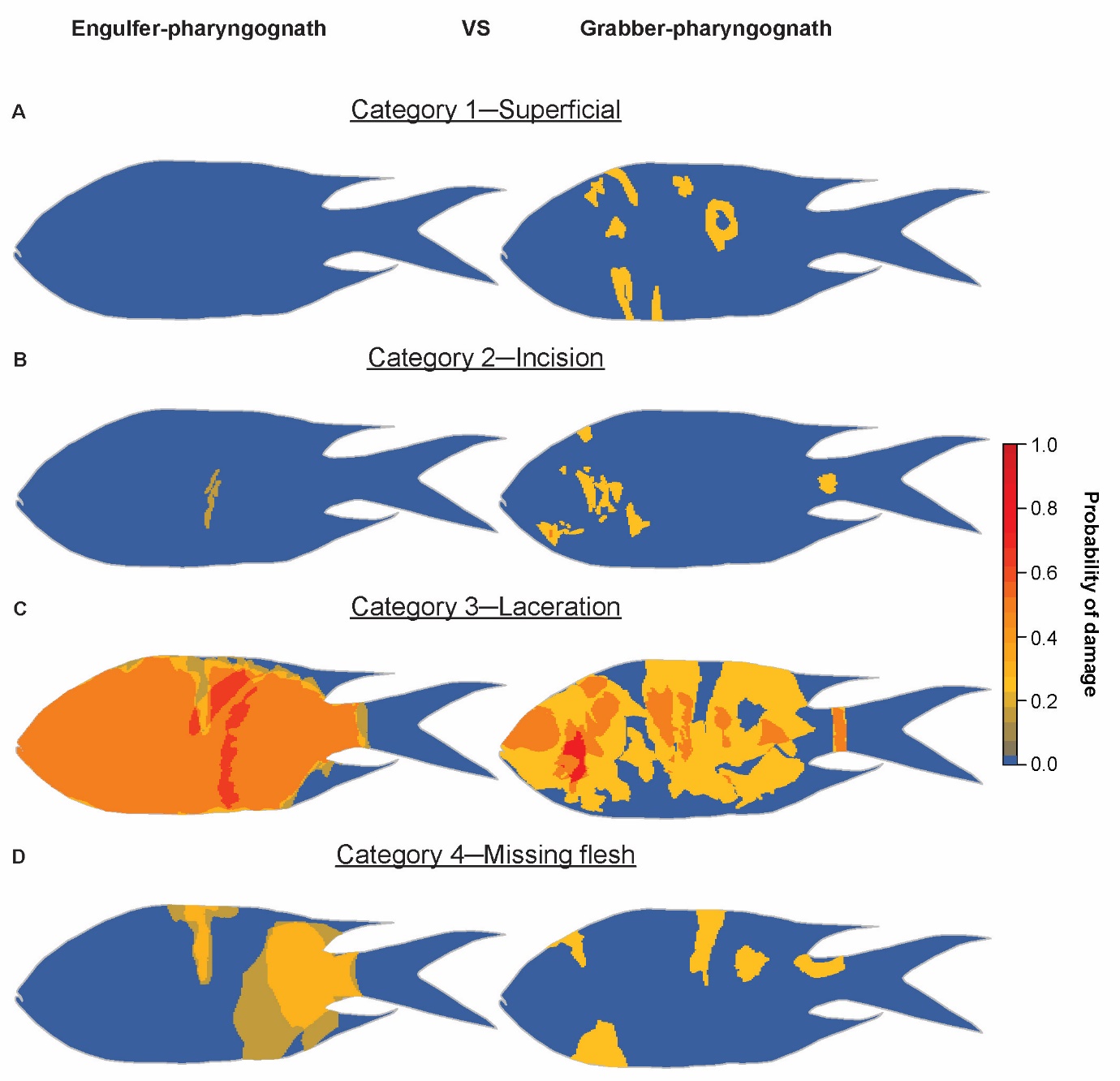
**

**Fig. S2** Heatmaps showing the probability of occurrence of four damage categories in any specific area on prey fish in engulfer-pharyngognath and grabber-pharyngognaths. (**A**) Superficial, (**B**) Incision (**C**) Laceration, and (**D**) Missing flesh.

**References**

Dean MN, Wilga CD, Summers AP. 2005. Eating without hands or tongue: Specialization, elaboration and the evolution of prey processing mechanisms in cartilaginous fishes. Biol Lett 1:357–61.

Ferguson AR, Huber DR, Lajeunesse MJ, Motta PJ. 2015. Feeding performance of King Mackerel, *Scomberomorus Cavalla*. J Exp Zool Part A Ecol Genet Physiol 323:399–413.

Grubich JR, Rice AN, Westneat MW. 2008. Functional morphology of bite mechanics in the great barracuda (*Sphyraena barracuda*). Zoology 111:16–29.

Holzman R, Day SW, Mehta RS, Wainwright PC. 2008. Jaw protrusion enhances forces exerted on prey by suction feeding fishes. J R Soc Interface 5:1445–57.

Juanes F, Conover DO. 1994. Piscivory and prey size selection in young-of-the-year bluefish: Predator preference of size-dependent capture success? Mar Ecol Prog Ser 114:59–70.

Mihalitsis M, Bellwood DR. 2017. A morphological and functional basis for maximum prey size in piscivorous fishes. PLoS One 12.

Mihalitsis M, Bellwood DR. 2019. Functional implications of dentition-based morphotypes in piscivorous fishes. R Soc Open Sci 6.

Motta PJ. 1984. Mechanics and functions of jaw protrusion in teleost fishes: a review. Copeia 1–18.

Motta PJ. 2004. Prey capture behavior and feeding mechanics of elasmobranchs. In: Biology of sharks and their relatives CRC Press Boca Raton. p. 165–202.

Oufiero CE, Holzman RA, Young FA, Wainwright PC. 2012. New insights from serranid fishes on the role of trade-offs in suction-feeding diversification. J Exp Biol 215:3845–55.

Reimchen TE. 1991. Evolutionary attributes of headfirst prey manipulation and swallowing in piscivores. Can J Zool 69:2912–16.

Westneat MW. 1994. Transmission of force and velocity in the feeding mechanisms of labrid fishes (Teleostei, Perciformes). Zoomorphology 114:103–18.

Whitenack LB, Motta PJ. 2010. Performance of shark teeth during puncture and draw: Implications for the mechanics of cutting. Biol J Linn Soc 100:271–86.
